# Effects of web platform delivery of a physical activity program for breast cancer survivors: a randomized controlled trial

**DOI:** 10.1007/s11764-025-01811-7

**Published:** 2025-05-27

**Authors:** Bernardine M. Pinto, Madison Kindred, Shira Dunsiger, Sheryl Mitchell, Ashwin Patel, Danielle Ostendorf, Amy G. Huebschmann

**Affiliations:** 1 College of Nursing, University of South Carolina, 1601 Greene Street, Room 302B, Columbia, SC 29208 USA; 2 Department of Kinesiology, Augusta University, Augusta, GA USA; 3 School of Public Health, Brown University, Providence, RI USA; 4 Pyx Health, Tucson, AZ USA; 5 Department of Medicine, University of Colorado, Anschutz Medical Campus, Aurora, CO USA; 6 Department of Kinesiology, Recreation, and Sports Studies, University of Tennessee, Knoxville, TN USA

**Keywords:** Breast cancer survivors, Physical activity, Web delivery, Randomized clinical trial

## Abstract

**Purpose:**

We previously demonstrated that a 3-month peer-delivered program (Moving Forward Together, MFT) significantly increased breast cancer survivors’ moderate-to-vigorous PA (MVPA). To enhance MFT’s scalability and reach, we adapted it to an existing web platform and developed webMFT. Our goal was to test the efficacy of webMFT on survivors’ MVPA.

**Methods:**

In a randomized controlled trial, we trained ten peer coaches from cancer care organizations to deliver webMFT or MVPA Tracking to 61 breast cancer survivors (mean age = 58.10 years [SD = 8.55], 1.40 years post-diagnosis [SD = 0.50], 80% Stage 0–1 cancer) for 3 months. Both groups received a FitBit® tracker and behavioral supports of weekly synchronization reminders, physical activity (PA) tipsheets, and recommended PA goals. In addition, webMFT participants received weekly coaching calls tailored to their FitBit® data shared through the web platform. All participants wore an Actigraph accelerometer for 7 days at baseline and at post-intervention and completed quality of life (QOL), mood, fatigue and physical functioning questionnaires. We used mixed effects regression models to examine between-group differences on outcomes.

**Results:**

Both groups significantly increased their MVPA from baseline to 12 weeks but there were no significant between-group differences in change in MVPA (*b* = − 22.84, SE = 16.99, *p* = .18). There were significant between-group effects favoring webMFT in improved QOL at 12 weeks (*b* = 1.56, SE = 0.77, *p* = .04).

**Conclusions:**

Adapting the efficacious MFT intervention for web delivery did not result in significant improvements in MVPA vs. MVPA Tracking. This raises questions as to whether the efforts undertaken to adapt and deliver the program through the web platform were justified as compared to MVPA Tracking with behavioral supports.

**Implications for Cancer Survivors:**

Promoting PA does not require web delivery of coaching—using physical activity trackers with weekly reminders and resources is also effective.

**Trial registration:**

This trial was registered in Clinicaltrials.gov on 6/8/2022 (NCT05409664).

## Background

With the increased evidence of the benefits of physical activity (PA) for individuals diagnosed with cancer, national guidelines have encouraged survivors to achieve at least 150 min/week of moderate-to-vigorous intensity PA (MVPA) [1, 2]. Breast cancer survivors are the largest subgroup of cancer survivors, approximately four million in the USA [3]. There are many programs available—ranging from on-site supervised, home-based and hybrid—that have been offered to support breast cancer survivors’ adoption of MVPA.

Peer support has been effectively used to help patients with diabetes, chronic kidney disease, and other chronic conditions manage their disease [4–6]. Peer support can increase the scale and reach of programs while costing less than programs delivered by research staff or healthcare professionals. Many cancer care organizations in the community offer survivors peer support delivered via phone or text/email contact. There are opportunities to promote MVPA utilizing peer mentors/coaches to reach survivors, thereby improving survivors’ well-being as well as expanding the scope of services that cancer care organizations offer.

We developed and tested a theory-based, home-based PA program (Moving Forward) for cancer survivors, delivered via 12 weekly calls from research staff. The program was effective in increasing MVPA among cancer survivors [7]. We subsequently partnered with the American Cancer Society’s peer support program, Reach to Recovery (consisting of breast cancer survivors who are trained to offer emotional and informational support to other survivors), to develop and test a peer-delivered version of Moving Forward called Moving Forward Together (MFT) [8]. We recruited, trained and supervised peer coaches (Reach to Recovery volunteers) to deliver MFT and demonstrated that survivors who received the program significantly increased MVPA vs. contact control [9]. The theory-based MFT program consisted of 12 weekly calls from the coach to problem solve and help the survivor to increase MVPA (based on the participant’s motivational readiness [10, 11]) and achieve 150 min/week of MVPA performed in bouts of at least 10 min in duration at 12 weeks. Participants wore a pedometer and heart rate monitor during their planned aerobic activity (primarily walking), maintained PA logs and reported their PA to the coach during weekly calls. Participants also received 12 PA tipsheets on topics such as safety, setting PA goals, and making PA enjoyable [8, 9].

To scale up this intervention, for the present study, we partnered with InquisitHealth (now, Pyx Health) to adapt their mentoring web platform—Mentor1 to1™—for MFT, thus creating “webMFT.” InquisitHealth, Inc. was a technology company that offered peer mentoring for chronic disease management via a web platform. The advantages of the web platform included (a) ability to customize coach-survivor matching criteria; (b) integration of counseling questions that coaches ask survivors during each call and to capture data entry for coaches, and (c) integration of activity tracker data to auto-capture MVPA, heart rate and step data. The matching criteria, progression in the 12-week program, counseling content and review of the survivor’s weekly MVPA data were based on our prior work [7–9].

In the first phase of the study, the Mentor1 to1™ web platform was modified to address (a-c) as described above. To ensure navigational ease and functionality robustness, the web platform was rapidly and iteratively tested by InquisitHealth’s team by conducting individual semi-structured interviews with four coaches (via videoconference) who had previously delivered MFT on their experience using the web platform. The web platform was then modified and user feedback from four additional coaches (with prior experience delivering MFT) was obtained during video-conferenced individual interviews.

Before implementing webMFT on a wider scale, we first wanted to test the efficacy of the web platform delivery and begin designing for the dissemination and implementation of webMFT [12]. The goals of this study were to first test the efficacy of webMFT in a randomized controlled trial on survivors’ accelerometer-measured MVPA at 12 weeks (post-intervention). Our hypothesis was that the group that received webMFT would significantly increase MVPA performed in bouts of at least 10 min in duration (“bouted MVPA”) vs. a comparison group (MVPA Tracking). Secondary outcomes included self-reported quality of life (QOL), mood, fatigue and physical functioning. The hypotheses were that the webMFT group would report improved QOL, mood and physical functioning and less fatigue vs. MVPA Tracking at post-intervention. We also planned to conduct descriptive analyses of the number and mean duration of calls delivered via the web platform. Finally, we determined a priori that webMFT would be judged feasible if > 75% of scheduled calls were delivered and participant satisfaction exceeded 80% on rating scales, at the end of the study (ratings of “moderately” to “very satisfied/acceptable”).

## Methods

### Design

This randomized controlled trial compared the effects of utilizing a web-based platform to deliver a 3-month theory-based PA program (webMFT) vs. MVPA Tracking among breast cancer survivors. Participants’ MVPA was assessed using an accelerometer at baseline and 3-months; secondary outcomes were assessed at the same timepoints. The study received approval from the Institutional Review Board at the University of South Carolina; the study was also registered (Clinicaltrials.gov NCT05409664).

#### Sample size and power analysis

Power calculations were based on prior work of this research team [9], as well as data from Cadmus-Bertram and colleagues [13] who compared web-based tracking plus Fitbit (FitBit® Inc, San Francisco, CA) to pedometer alone among post-menopausal women. Results from Cadmus-Bertram found minimal increases in accelerometer-measured MVPA from baseline to end of treatment in the Fitbit condition. Comparing the published prior effects of our MFT program [9] to web-based tracking plus Fitbit on changes from baseline in MPVA yielded a medium effect size, *d* = 0.60. Assuming similar effect sizes for the current study and a two-sided alpha = 0.05, we estimated that there would be sufficient power to test the effects of webMFT vs. MVPA Tracking on min/week of bouted MVPA at the end of intervention with 20 survivors/arm. However, given the risk of powering on pilot studies and the fact that Cadmus-Bertram and colleagues measured outcomes at 16 weeks (vs. 12 weeks in the current study) and allowing for 10% attrition, we conservatively inflated the sample size to *N* = 56 (28 survivors/arm).

### Coach recruitment and eligibility

Coaches were recruited from four cancer care organizations in the USA (Cancer Hope Network, Pink Lemonade Project, Todd Memorial Hospital and the Delaware Breast Cancer Coalition). For an organization to be eligible to partner with the study, they needed to have an established peer mentoring program. Research staff worked with organization staff members to share study flyers and brochures with their peer mentors. If a peer mentor was interested in participating in the study, she called the Research Assistant to complete the phone screen.

Coach eligibility included (1) current peer coach (at least 1 year) at the organization, (2) have an email address, access to a telephone, and owned a computer/tablet with internet access for the web platform, (3) willing to participate in online group training, (4) willing to have calls recorded and receive feedback from research staff, and (5) currently exercising for at least 60 min/week. If eligible at the phone screen, coaches were sent a technology quiz to determine their typing speed (a minimum of 20 words/min) and internet broadband strength so that they could utilize the platform. After completing the phone screen and technology quiz, coaches began training.

### Training coaches

Before training, coaches were sent a binder which included training materials, and presentation print outs. Coach training consisted of three components: (1) recorded modules, (2) group training to practice a mock call, and (3) individual training to practice a mock call while using the platform. Participants received a link to access recorded modules that were self-paced. The research team asked the coach to view the six recorded modules within a 2-week period. Each module was 20–30 min in duration and described the study design, Health Insurance Portability and Accountability Act (HIPAA) protections, and theoretical concepts relating to PA. After the coach viewed each module, she was asked to complete a comprehension quiz via REDCap (Research Electronic Data Capture, Vanderbilt University) [14, 15]. The final recorded module provided by InquisitHealth (length = 70 min) provided training on the web platform. The module covered topics such as how to schedule calls, how to locate an assigned participant on the web platform, how to enter data, and how to send information (i.e., tip sheets) to the participant. Coaches were provided a test profile within the platform to practice using the platform and they were encouraged to practice on their own.

To prepare for the group training session, coaches listened to a pre-recorded mock call to understand the key components. The group training was held on Microsoft Teams; each group training session consisted of 3–4 coaches in addition to a research staff member and the PI. During the group session, each coach completed a mock call in which feedback was provided by the PI, research staff member, and other coaches. The final training component consisted of an individual virtual meeting with a research staff member to complete a mock call while using the platform. The coach shared her screen with the staff member to ensure proper execution of delivery. The research staff served as the mock participant. A checklist of key intervention tasks (navigating the platform, data entry, scheduling) was used to ensure each coach was comfortable using the platform to deliver the intervention. Once training was complete and the coach felt comfortable with intervention delivery, the coach was eligible to be paired with a participant. Coaches were provided booster sessions (if needed) prior to being paired with a participant if there was a significant amount of time between training and participant-mentor matching.

### Participants

Women were eligible if they were (1) ≥ 21 years of age, (2) diagnosed with Stage 0–3 breast cancer within the last 5 years, (3) able to read and speak English, (4) ambulatory and able to walk unassisted, (5) sedentary (< 30 min/week of self-reported vigorous, or < 90 min/week of moderate intensity PA in the past 6-months), and (6) owned a smartphone with Bluetooth capabilities. Women with medical or psychiatric problems (e.g., myocardial infarction, stroke, transient ischemic attacks, substance abuse, and orthopedic problems) that might interfere with protocol adherence were excluded.

### Participant recruitment

Survivors were recruited from partner cancer care organizations, a local oncology practice, a hospital tumor registry, and breast cancer support groups. Staff members from the organizations sent a study invitation and study flyers through their email list servs. A letter and a study brochure were mailed to the names on the tumor registry. If a survivor was interested in participating, she was asked to call the Research Assistant to complete an initial phone screen. Upon completion of the phone screen, participants were sent an electronic informed consent to review and sign. After the informed consent was received, a medical consent was sent to the patient’s oncologist. Then, participants were mailed an Actigraph GT3X accelerometer to obtain baseline PA data. Participants also received questionnaires to complete via REDCap. Upon successful confirmation and receipt of Actigraph data over 7 days, confirmation of a sedentary lifestyle (defined as < 30 min/week of bouted MVPA), and completion of the questionnaires, participants were mailed a Fitbit Inspire 2 (FitBit® Inc, San Francisco, CA) in addition to their research username and password to login to Fitbit.

### Randomization

Participants were randomized to the webMFT intervention or MVPA Tracking. The sample was stratified for two variables, age (< 60 years, or ≥ 60 years) and treatment (received chemotherapy, not received). After the participant received her Fitbit and set up the device using the designated username and password, she was asked to contact the Project Coordinator to be randomized.

### Intervention groups

After the baseline assessments were completed and the participant successfully received and logged in to her research Fitbit account, the Project Coordinator opened a sealed envelope indicating group assignment and contacted the participant regarding group assignment via telephone. If the participant was assigned to the webMFT group, a profile was created in the web platform, and she was matched with a coach. All participants received a Fitbit Inspire 2 and were asked to wear the tracker (on the nondominant wrist) daily for 3 months. Both groups received PA goals to gradually increase their activity up to 150 min of MVPA, in addition to 12 tip sheets that covered topics such as exercising safely and exercising in different types of weather.

### webMFT group

Coaches used the web platform to deliver the intervention, accept coaching requests, initiate secure phone calls, send text messages, and view/send participants their Fitbit data. After the coach was matched with a participant, she viewed a brief profile of the new participant. Prior to a scheduled call, the coach viewed the Fitbit data of the participant on the web platform and sent the data to the participant. Then she called the participant via the platform (the calls were automatically recorded on the platform) and began the PA counseling call, which included responding to each intervention prompt such as review of the participant’s weekly Fitbit data (active minutes), set goals, discuss barriers to PA, and assess any symptoms related to PA such as chest pain during PA. Counseling calls were grounded in the Transtheoretical Model and Social Cognitive Theory [10, 16]. Coaches entered the participant responses directly into the web platform. Coaches discussed the weekly tip sheets with their participants and sent the tip sheet to the participant via text or email, per participant preference. Following each call, coaches were asked to complete a post-call checklist to ensure all data was properly entered and the next call was scheduled. To promote fidelity, the Project Coordinator reviewed each recorded call and provided bi-weekly feedback individually to the coaches.

Research staff sent weekly text reminders to webMFT participants to ensure the Fitbit was consistently syncing with the Fitbit app. This ensured the Fitbit activity data (active minutes which is the sum of very active minutes and fairly active minutes) were pulled into the platform so that the coach could review the participant’s PA data before calling the participant.

### MVPA Tracking group

Participants in this group also received a Fitbit Inspire 2 tracker, however they did not receive coach calls. They did receive the weekly activity goals, which outlined a gradual progression of activity up to 150 min of MVPA in addition to the 12 tip sheets. MVPA Tracking participants also received the same weekly text reminders from the research team as the webMFT group, to ensure they were consistently syncing their Fitbit.

### Quality control

The Project Coordinator reviewed all calls through the platform on a weekly basis; feedback was provided to each coach individually during biweekly supervisory calls. The PI audited 10% of calls on a monthly basis to ensure coaches were adhering to the intervention protocol (content and counseling process).

### Measures

Demographic (age, ethnic group, education, etc.) information as reported by the participant, and medical and treatment history, provided by the health care provider (e.g., participant’s cancer stage, date of diagnosis) were collected at baseline. Participants were mailed an Actigraph GT3X accelerometer to wear for 7 days to obtain baseline PA data. The primary outcome was accelerometer-measured bouted MVPA and secondary outcomes included QOL, mood, fatigue, and physical functioning assessed at baseline and 12 weeks.

#### Actigraph GT3X accelerometer

Participants were asked to wear the Actigraph accelerometer (GT3X, Penscola, FL) on their dominant hip for 7 days, excluding bathing, swimming, and sleeping. Participants were asked to wear the Actigraph for a minimum of 10 h per day and were required to have 4 valid days of wear. Freedson adult cut-points [17] were used to classify different intensities of PA and to calculate bouted MVPA (at least 10 min) and total MVPA. The data was collected in 10-s epochs. Participants wore the Actigraph at baseline and 12 weeks.

#### Quality of life (QOL)

QOL was assessed using a 37-item questionnaire, the Functional Assessment of Cancer Therapy Scale-Breast (FACT-B) [18]. FACT-B assessed physical well-being, social well-being, emotional well-being, functional well-being, and included a breast cancer subscale. Participants rated five domains of their QOL from 0 (not at all) to 4 (very much). The higher the score, the better the QOL [18]. The total score and the Breast Cancer Symptom subscale were analyzed.

#### Mood

The Profile of Mood States (POMS) 65-item questionnaire was used to assess an individual’s mood during the past week [19]. The participants rated how they felt on 6 mood dimensions (e.g., tension, anxiety, depression) on a 5-point Likert scale ranging from 0 (not at all) to 4 (extremely). A total mood disturbance (TMD) score (− 30 to 200) was calculated using all subscales. Greater mood disturbance was indicated by higher TMD scores, while a lower TMD score indicated less mood disturbance [19].

#### Fatigue

The Functional Assessment of Chronic Illness Therapy Fatigue Scale (FACIT-F) consists of 13 items and was used to assess self-reported fatigue and its impact on daily activities. The participants rated their fatigue from 0 (not at all) to 4 (very much). Higher scores indicated less interference of fatigue on daily activities [20].

#### Physical functioning

The 36-item Short Form Health Survey (SF- 36) assesses 8 health concepts: physical functioning, role limitations- physical health, bodily pain, social functioning, mental health, role-limitations-emotional problems, vitality, and general health perceptions [21]. We analyzed the SF- 36 Physical Functioning Scale (10 items). Higher scores indicated the ability to perform various physical activities such as walking a half mile.

#### End of study evaluation

Study participants completed an evaluation of the program and its components at the end of the study. A single question was used to assess participants’ satisfaction with the program. The participants rated their satisfaction from 1 (not at all) to 5 (very satisfied). Higher scores indicated greater satisfaction with the program.

### Analyses

Descriptive statistics were used to summarize both the participant and coach samples with respect to baseline socio-demographics, and medical health variables. Between-group differences (webMFT vs. MVPA Tracking) in baseline characteristics were assessed using *t*-test (for continuous variables) and chi-squared tests for categorical variables.

A mixed effects regression model was used to examine between-group differences (webMFT vs. MVPA Tracking) on primary and secondary study outcomes. First, we regressed min/week of bouted MVPA collected via accelerometer at 12 weeks on baseline bouted MVPA, group (webMFT vs. MVPA Tracking) and accelerometer wear time. The model was specified to include a random intercept to adjust for repeated measures within participant over time. Although we explored clustering by coach, there were no effects, and thus, this was removed from the final model. A similar approach was used to estimate between-group differences in secondary outcomes (QOL, mood, fatigue, and physical functioning) over 12 weeks. As proposed, we also analyzed total MVPA from the Actigraph device, as well as active minutes from the Fitbit collected over 12 weeks from participants in both groups. Analyses were conducted on the Intent-to-Treat sample; thus, models included all randomized participants regardless of the amount of data they contributed. Mixed effects models use a likelihood-based approach to estimation and do not require any direct imputation of missing outcomes.

Finally, feasibility and acceptability of webMFT were assessed via objectively measured number and duration of intervention calls tracked on the web platform, and participant satisfaction was reported on the end of the study evaluation.

Analyses were conducted in R Studio 3.6.0 [22] with significance level set at 0.05 a priori.

## Results

### Sample

**Fig. 1:**
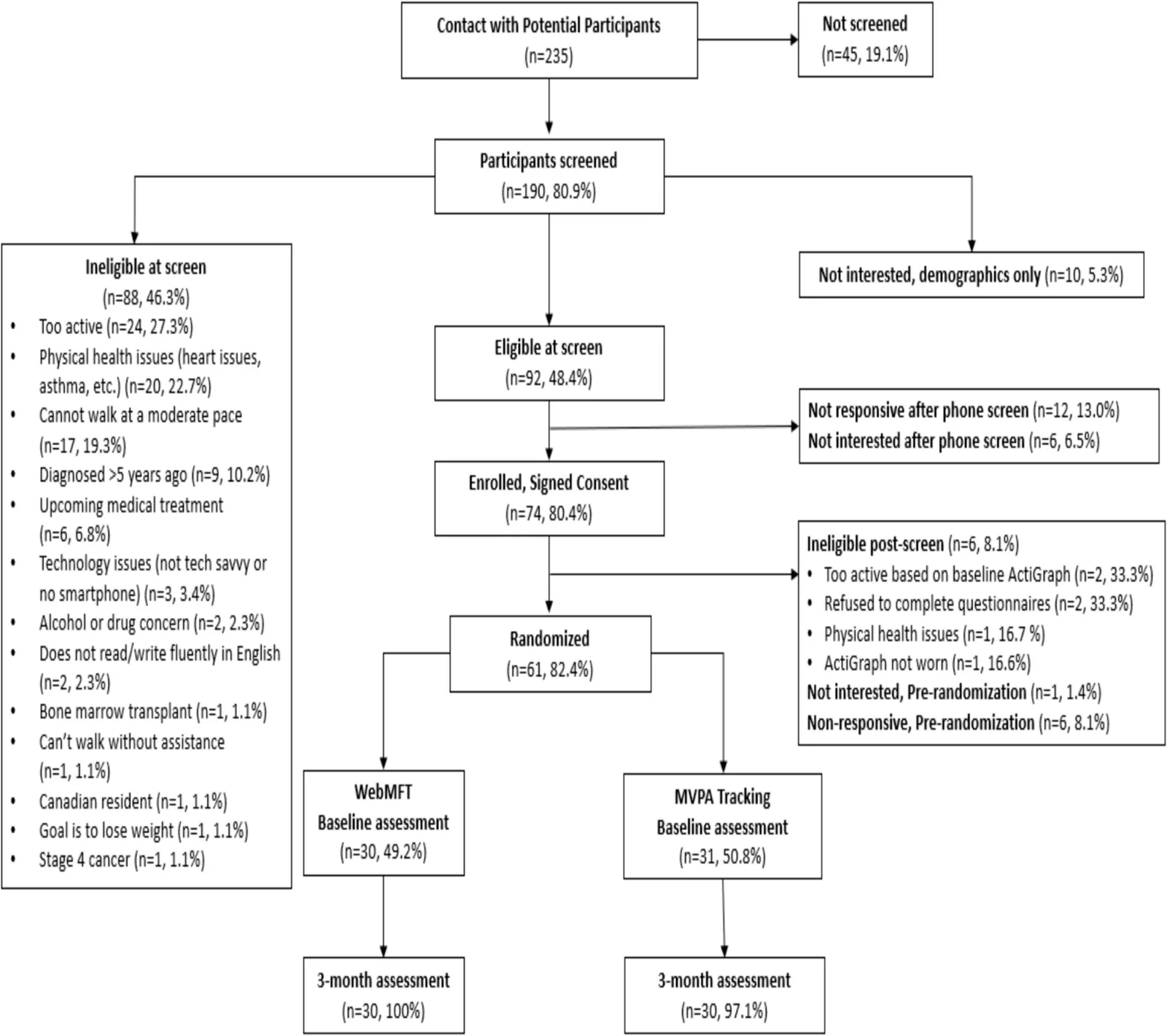
CONSORT diagram of participant recruitment

A total of 235 participants were approached for screening, of which 190 (81%) were screened for eligibility. Of those, 92 (49%) screened eligible and 74 (80%) enrolled in the study. Six participants (8%) were classified as ineligible post-enrollment, yielding a final randomized sample of 61 (or 82% of those who screened eligible). See Fig. 1 for complete details.

A full description of the study sample (*N* = 61) is presented in Table 1. Participants were 57.57 years of age on average (SD = 11.73), predominantly White/Caucasian (92%) and self-reported their ethnicity as Not Hispanic or Latino (95.1%). The sample was highly educated (90% had at least some college level education). There were no significant between-group differences (webMFT vs. MVPA Tracking) in baseline socio-demographics, or medical history variables (all *p*-values > 0.05). All participants provided data at baseline and 12 weeks (one participant did not provide valid Actigraph data at 12 weeks).

**Table 1:** Baseline descriptives by study arm (*N* = 61)

	webMFT *N* = 30	MVPA Tracking *N* = 31	Overall, *N* = 61	*p-value*
Age, years	58.10(8.55)	57.06(14.29)	57.57(11.73)	0.73
Gender (women)	30(100%)	31(100%)	61(100%)	0.99
Race				0.34
White/Caucasian	24(80%)	26(83.9%)	50(92%)	
Black/African American	4(13.3%)	5(16.1%)	9(14.8%)	
Other	2(6.7%)	0	2(3.3%)	
Ethnicity				0.07
Not Hispanic or Latino	27(90%)	31(100%)	58(95.1%)	
Hispanic or Latino	3(10%)	0	3(4.9%)	
Education				0.94
HS diploma	3(10%)	2(6.5%)	5(8.2%)	
Vocation/trade school	0	1(3.2%)	1(1.6%)	
Some college	5(16.7%)	6(19.4%)	11(18%)	
Associate degree	4(13.3%)	4(12.9%)	8(13.1%)	
Bachelor’s degree	8(26.7%)	8(25.8%)	16(26.2%)	
Graduate school	10(33.3%)	10(32.3%)	20(32.8%)	
Employment				0.18
Full time	17(56.7%)	14(45.2%)	31(50.8%)	
Part time	3(10%)	0	3(4.9%)	
Retired	8(26.7%)	12(38.7%)	20(32.8%)	
Homemaker	2(6.7%)	3(9.7%)	5(8.2%)	
Unemployed	0	2(6.5%)	2(3.3%)	
Time since diagnosis, years	1.40(0.50)	1.35(0.49)	1.38(0.49)	0.72
Cancer stage				0.57
Stage 0	5(16.7%)	2(6.5%)	7(11.5%)	
Stage 1	19(63.3%)	21(67.7%)	40(65.6%)	
Stage 2	4(13.3%)	4(12.9%)	8(13.1%)	
Stage 3	2(6.7%)	4(12.9%)	6(9.8%)	
Surgery				
Lumpectomy	15(50%)	20(64.5%)	35(57.4%)	0.25
Mastectomy	8(26.7%)	4(12.9%)	12(19.7%)	0.18
Treatment				
Chemotherapy	15(50%)	14(46.7%)	29(48.3%)	0.78
Radiation	22(95.7%)	21(100%)	43(97.7%)	0.33
Hormone therapy	25(83.3%)	23(74.2%)	48(78.7%)	0.30

*N*(%) for categorical variables; mean(standard deviation) for continuous variables; *p*-value for between-group difference

### Coaches

A total of 28 interested coaches completed the phone screen and tech questions, 15 were deemed eligible (54%). Eleven coaches completed the training and 10 were paired with a participant(s). Coach characteristics are presented in Table 2. Similar to the participant sample, coaches were 57.60 years of age on average (SD = 7.78), predominantly White/Caucasian (90%) and well-educated (at least some college level education or higher).

**Table 2:** Descriptives of coach sample

	*N* = 10
Age, years	57.60(7.78)
Gender(women)	10(100%)
Race	
White/Caucasian	9(90%)
Black/African American	0
Other	1(10%)
Ethnicity	
Not Hispanic or Latino	10(100%)
Hispanic or Latino	0
Education	
HS diploma	0
Vocation/trade school	0
Some college	1(10%)
Associate degree	0
Bachelor’s degree	5(50%)
Graduate school	4(40%)
Employment	
Full time	6(60%)
Part time	0
Retired	3(30%)
Homemaker	0
Unemployed	0
Medical leave	1(10%)
Time since diagnosis, years	8.44(4.22)
Cancer stage	
Stage 1	4(40%)
Stage 2	4(40%)
Stage 3	1(10%)
Stage 4	1(10%)

### Physical activity outcomes

Unadjusted baseline rates of PA (as measured by the Actigraph) are presented in Table 3. Participants randomized to webMFT significantly increased their bouted MVPA (min/week) from 4.06 (SD = 9.36) min/week at baseline to 31.68 (SD = 44.86) min/week at 12 weeks, *p* < 0.001. Similarly, MVPA Tracking participants increased their bouted MVPA (min/week) from 5.02 (SD = 12.05) min/week at baseline to 56.03 (SD = 84.42) min/week at 12 weeks, *p* < 0.001. There were no significant between-group differences in change in bouted MVPA from baseline to 12 weeks (*b* = − 22.84, SE = 16.99, *p* = 0.18). Similarly, both groups significantly increased their total MVPA (min/week) (webMFT: from 111.61 [SD = 71.59] to 165.64 [SD = 75.57]; MVPA Tracking: from 128.81 [SD = 86.07] to 198.54 [SD = 150.35]), but there were no significant between-group differences in change in total MVPA (min/week) from baseline to 12 weeks (*b* = − 14.59, SE = 22.02, *p* = 0.51).

**Table 3:** Changes in min/week of accelerometer-measured MVPA by group

	webMFT	MVPA Tracking	Between-group effect	*p*-value
Min/week MVPA				
Baseline	4.06(9.36)	5.02(12.05)	− 22.84(16.99)	0.18
12 weeks	31.68(44.86)	56.03(84.42)		

Between-group effect is an estimated regression parameter b with standard error, corresponding to absolute difference at 12 weeks adjusting for baseline and accelerometer wear time. *p*-value examines the significance of b. Models adjust for baseline-value

### Secondary outcomes

Secondary outcomes by group are presented in Table 4. Results indicate significant between-group effects favoring webMFT in changes in QOL (Fact-B total score) over time. Specifically, those randomized to webMFT reported significantly greater improvements in QOL over 12 weeks compared to MVPA Tracking participants (*b* = 1.56, SE = 0.77, *p* = 0.04). Although not significant, point estimates were in the expected direction for breast cancer symptoms, mood, fatigue and physical functioning.

**Table 4:** Changes in secondary outcomes over time by group

	webMFT	MVPA Tracking	Between-group effect	*p*-value
Quality of life (QOL)				
Baseline	98.66(26.32)	106.72(17.84)		
12 weeks	113.13(19.03)	113.76(17.02)	1.56(0.77)	0.04*
Breast cancer symptom index (BCS)				
Baseline	22.63(7.31)	24.26(5.14)		
12 weeks	25.20(5.60)	25.71(5.76)	0.48(1.09)	0.65
Mood (total mood disturbance)				
Baseline	51.40(11.31)	48.91(8.51)		
12 weeks	49.66(8.73)	50.33(11.23)	− 1.98(2.23)	0.38
Fatigue				
Baseline	30.47(11.86)	36.84(10.34)		
12 weeks	40.70(10.07)	42.23(7.59)	0.47(2.20)	0.83
Physical functioning (SF- 36)				
Baseline	79.17(14.80)	78.87(18.01)		
12 weeks	85.50(13.15)	84.84(11.51)	0.56(2.87)	0.85

Between-group effect is an estimated regression parameter b with standard error, corresponding to absolute difference at 12 weeks. *p*-value examines the significance of b. Models adjust for baseline-value. *p < 0.05. QOL- FACT-B (higher = better QOL). BCS Index -Breast Cancer Symptom Index (higher = less symptom burden). Mood -POMS (higher = greater mood disturbance). Fatigue – FACT-F (higher = less impact of fatigue), physical functioning – SF36 (higher = better)

**Fig. 2:**
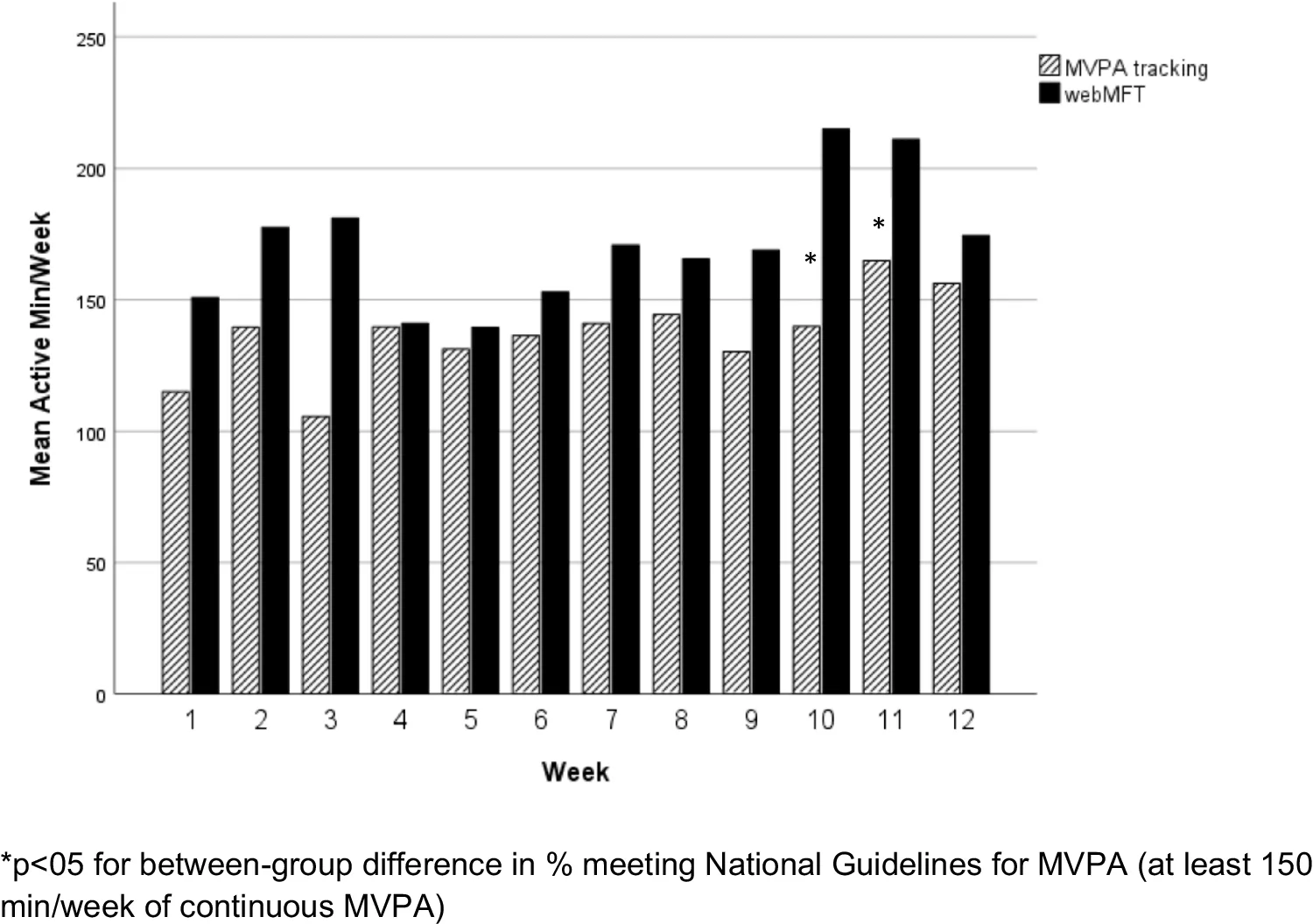
Weekly active minutes from the Fitbit by study arm over 12 weeks

When considering Fitbit data, there were significant correlations between steps/week and bouted MVPA (from Actigraph): *r* = 0.55, *p* < 0.001 and significant correlations between Fitbit active minutes and Actigraph-measured bouted MVPA, *r* = 0.52, *p* < 0.001. Although not significant, between-group differences were in the expected direction with greater Fitbit active minutes for webMFT compared to MVPA Tracking at 12 weeks (*p* = 0.22). When considering change over time, webMFT participants went from a mean of 150.83 min/week at week 1(SD = 136.56) to a mean of 174.43 min/week (SD = 120.98) at 12 weeks compared to an increase from 114.90 min/week at week 1 (SD = 99.59) to 156.20 min/week at week 12 for MVPA Tracking (no significant between-group differences in change over time). Also, when comparing the weekly Fitbit active minutes by group across 12 weeks (Fig. 2), the webMFT group had a significantly higher prevalence of participants meeting the national guidelines for MVPA of at least 150 min/week at weeks 10 and 11 (*p* < 0.05 for between-group difference in percent meeting national guidelines for MVPA).

### Feasibility and acceptability

When considering acceptability of the intervention, 100% of webMFT participants reported satisfaction with the program (score of 3 or above on a scale of 1–5, with 1 = not at all satisfied and 5 = very satisfied) and 80% of participants reported being very satisfied. On average, 90% of calls were completed, indicating a feasible intervention by our pre-specified criteria. The average length of calls was 23.21 min (SD = 6.53; range, 14.17–42.18).

## Discussion

Contrary to our hypothesis, adapting the efficacious MFT intervention for delivery through an online platform as webMFT did not result in significant improvements in Actigraph-measured bouted MVPA, as compared to MVPA Tracking. In terms of secondary outcomes, only QOL effects were significantly greater in the webMFT group as compared to the MVPA Tracking group, with no significant differences observed in mood, fatigue, or physical functioning. Both study groups significantly improved bouted and total MVPA from baseline to 12 weeks (*p* < 0.05 pre-post for both groups). These findings raise questions as to whether the “juice is worth the squeeze” for the adaptations required to deliver MFT using a web-based platform. In other words, what was the impact of webMFT’s additions of theory-based PA coaching delivered from the online platform to the behavioral supports common to both the webMFT and MVPA Tracking groups (use of the Fitbit PA tracker, weekly reminders to sync the PA tracker, graded PA recommendations, and PA tip sheets)?

There are several methodologic measurement issues to consider when interpreting the MVPA findings. While the Fitbit active minutes, estimated from a wrist-worn device, and bouted MVPA, estimated from the hip-worn Actigraph, were moderately correlated at *r* = 0.55, the group differences varied between the two devices: a non-significant 24-min/week increase in bouted MVPA measured with the Actigraph device in the MVPA Tracking group vs. webMFT and a non-significant 18-min/week increase measured using Fitbit active minutes in MVPA Tracking vs. webMFT. Conversely, the webMFT group were more likely to meet the PA guidelines of 150 min/week of continuous MVPA at weeks 10–11 (Fig. 2) and also attained higher mean FitBit active minutes (174 min/week) as compared to the MVPA Tracking group (156 min/week) at 12 weeks. In general, prior studies typically report that Fitbit devices tend to overestimate steps and time spent in higher-intensity activities in free-living settings [23–25]. Limited studies exist that specifically compare the Fitbit Inspire 2 (model used in this study) to the Actigraph GT3X among adults. However, one recent study reported that the Fitbit Inspire 2 overestimated steps and minutes of light, moderate and MVPA (average of 18 min/day, range 30 to 128 min/day) as compared to the Actigraph GT3X-BT and that this bias increased as PA volume increased [26]. However, this study was conducted in a sample of youth with hemophilia, which may not be generalizable to adult survivors with breast cancer. Other reasons for these discrepancies in PA estimates from the two devices could have been due to differences in timing of when devices were worn, wear location (hip vs. wrist), data filtering algorithms, intensity cut-off equations, and data processing.

Beyond the statistical significance of these data, one must also consider clinical importance. Specifically, though there were no statistically significant between-group differences in either Actigraph-measured PA (primary outcome) or Fitbit active minutes, the mean Fitbit active minutes in the webMFT group exceeded the target of 150 min/week (mean 174 min at 12 weeks) while the mean Fitbit active minutes were closer to guidelines at 12 weeks for the MVPA Tracking group (mean 156 min at 12 weeks). If one considers that “you can’t manage what you can’t measure,” participants in both study groups and coaches in the webMFT group were tracking Fitbit active minutes to inform their weekly goal setting and counseling, so one would expect a stronger effect size between groups on the outcome of Fitbit active minutes than by Actigraph. Although we powered this study based on a Cohen’s d effect size of 0.60 based on prior work showing minimal improvement in PA by using PA trackers alone [13], other behavioral prompts delivered to the MVPA Tracking group may have led to a stronger benefit in this “comparison” group than expected. Specifically, those behavioral prompts included the weekly reminders to sync the tracker, the tip sheets, graded recommendations to achieve 150 min weekly and proprietary Fitbit PA tracker “nudges” to user’s smart phone devices; these fulfill several “behavior change techniques” [27] including regular tracking, feedback on progress, and external accountability. Accordingly, these behavior change techniques for the MVPA Tracking group may have yielded greater improvements in this comparison arm than were expected from the prior studies of MFT [9, 28] and the earlier published Fitbit PA tracker work (weekly email updates of PA progress rather than regular “nudges” to smartphone Fitbit mobile apps). Importantly, participants in the MVPA Tracking group demonstrated significant improvements in bouted MVPA, indicating that provision of a Fitbit device, suggested weekly PA goals and sync reminders were effective at increasing MVPA levels. This is exciting because it is a low-cost, low-touch approach that is scalable. However, we were unable to examine whether these increases were maintained long-term (> 1 year) in the context of the present study, which is a limitation. Nonetheless, peer mentoring may still offer benefits for those survivors who are isolated due to medical comorbidities/immunocompromised or, living in rural settings and need support that goes beyond the program received by the MVPA Tracking group.

The significant improvement in QOL in the webMFT group is consistent with our prior findings. In the absence of significant group differences in either MVPA or physical function, we speculate that this improvement in QOL may be related to the social support provided by the webMFT coaches as part of their webMFT weekly counseling calls that the MVPA Tracking group did not receive. The webMFT group showed an improvement of 14.47 points on the FACT-B (MVPA Tracking group improved by 7.04 points) exceeding the minimally important difference of 7–8 points [29].

As a type 1 hybrid implementation-effectiveness trial, this study also evaluated feasibility and acceptability outcomes. The webMFT group met the pre-specified thresholds for feasibility (completed 90% of calls) and acceptability was very high (100% satisfied/80% extremely satisfied). These implementation outcomes demonstrate that the lack of statistically significant difference in MVPA was not due to lack of engagement with the program by participants. As mentioned at the outset of the Discussion, one must ask if the “juice was worth the squeeze?” to adapt MFT to a web-based platform to deliver webMFT. In our prior work, MFT had a stronger effect size to increase MVPA when it was simpler—no web platform for coaches to navigate, and when it was compared to a control group that included fewer behavioral change techniques (the comparison arm received weekly calls assessing health symptoms and survivorship tipsheets from the peer coach) [9]. The use of the web platform to deliver MFT and the enhanced effects of the Fitbit in the MVPA Tracking group may partly explain the findings of our current study.

This study had several strengths and notable limitations. The strengths included that the participant retention was high, the study exceeded accrual goals, and both the webMFT and MVPA Tracking study groups used commercially available Fitbit trackers that are widely available for replicating findings. Limitations included that the study participants comprised a well-educated, and largely racially homogeneous, white sample. Another limitation is that some coaches expressed difficulties navigating the web platform, and this may have attenuated their focus from the intervention content material.

## Conclusion

While QOL effects were significantly greater in the webMFT vs. MVPA Tracking and both groups significantly improved MVPA—these results raised questions as to whether extra effort to develop webMFT (and train coaches to use webMFT) was worth it as compared to MVPA Tracking alone by a Fitbit PA tracker with the additional behavioral supports provided in this study. A question remains as to whether the simpler “original” MFT with fewer “bells and whistles” may actually outperform webMFT, as webMFT had a more limited effect size than prior published data of MFT [9, 28]. Future work in this line of inquiry may benefit from considering multi-phase optimization study approaches to compare the presence/absence of a Fitbit PA tracker, and MFT with or without a web platform on outcomes of Fitbit active minutes and accelerometer measured MVPA and related psychosocial outcomes.

## Data Availability

The datasets (de-identified) analyzed during the current study are available from the corresponding author on reasonable request.
